# 13q Deletion Syndrome Involving *RB1*: Characterization of a New Minimal Critical Region for Psychomotor Delay

**DOI:** 10.3390/genes12091318

**Published:** 2021-08-26

**Authors:** Flavia Privitera, Arianna Calonaci, Gabriella Doddato, Filomena Tiziana Papa, Margherita Baldassarri, Anna Maria Pinto, Francesca Mari, Ilaria Longo, Mauro Caini, Daniela Galimberti, Theodora Hadjistilianou, Sonia De Francesco, Alessandra Renieri, Francesca Ariani

**Affiliations:** 1 Medical Genetics, University of Siena, 53100 Siena, Italy; flavia.privitera@dbm.unisi.it (F.P.); gabriella.doddato@dbm.unisi.it (G.D.); filomena.papa@dbm.unisi.it (F.T.P.); margherita.baldassarri@dbm.unisi.it (M.B.); francesca.mari@unisi.it (F.M.); alessandra.renieri@unisi.it (A.R.); 2 Med Biotech Hub and Competence Center, Department of Medical Biotechnologies, University of Siena, 53100 Siena, Italy; 3 Unit of Pediatrics, Department of Maternal, Newborn and Child Health, Azienda Ospedaliera Universitaria Senese, Policlinico ‘Santa Maria alle Scotte’, 53100 Siena, Italy; arianna.calonaci@student.unisi.it (A.C.); mauro.caini@ao-siena.toscana.it (M.C.); d.galimberti@aou-siena.toscana.it (D.G.); 4 Genetica Medica, Azienda Ospedaliera Universitaria Senese, 53100 Siena, Italy; annamaria.pinto@dbm.unisi.it (A.M.P.); Longo@unisi.it (I.L.); 5 Unit of Ophthalmology and Retinoblastoma Referral Center, Department of Surgery, University of Siena, Policlinico ‘Santa Maria alle Scotte’, 53100 Siena, Italy; dorisocularoncology@libero.it (T.H.); sonia.defrancesco@ao-siena.toscana.it (S.D.F.)

**Keywords:** 13q deletion syndrome, retinoblastoma, array-CGH, intellectual disability, NBEA

## Abstract

Retinoblastoma (RB) is an ocular tumor of the pediatric age caused by biallelic inactivation of the *RB1* gene (13q14). About 10% of cases are due to gross-sized molecular deletions. The deletions can involve the surrounding genes delineating a contiguous gene syndrome characterized by RB, developmental anomalies, and peculiar facial dysmorphisms. Overlapping deletions previously found by traditional and/or molecular cytogenetic analysis allowed to define some critical regions for intellectual disability (ID) and multiple congenital anomalies, with key candidate genes. In the present study, using array-CGH, we characterized seven new patients with interstitial 13q deletion involving *RB1*. Among these cases, three patients with medium or large 13q deletions did not present psychomotor delay. This allowed defining a minimal critical region for ID that excludes the previously suggested candidate genes (*HTR2A*, *NUFIP1*, *PCDH8*, and *PCDH17)*. The region contains 36 genes including *NBEA*, which emerged as the candidate gene associated with developmental delay. In addition, *MAB21L1*, *DCLK1*, *EXOSC8*, and *SPART* haploinsufficiency might contribute to the observed impaired neurodevelopmental phenotype. In conclusion, this study adds important novelties to the 13q deletion syndrome, although further studies are needed to better characterize the contribution of different genes and to understand how the haploinsufficiency of this region can determine ID.

## 1. Introduction

Retinoblastoma (RB) is the most frequent intraocular tumor of the pediatric age with an incidence of 1/15,000–28,000 live births [1]. It is caused by biallelic inactivation of the *RB1* oncosuppressor gene located in 13q14.2 [1]. Constitutive gross-sized deletions involving *RB1* are present in 10% of cases [2,3,4,5]. When the deletion involves part of the *RB1* surrounding genome, it causes a rare contiguous gene deletion condition known as 13q deletion syndrome [1]. The syndrome is characterized by RB, developmental anomalies, and peculiar facial features.

In 1983 the facial phenotype of patients with RB and 13q deletions was firstly described, observing three Japanese cases with prominent eyebrows, a broad nasal bridge, a bulbous tip of the nose, a large mouth with a thin upper lip, and a long prominent philtrum [6]. In a subsequent paper, Baud et al. [7] described 22 RB patients showing anteverted ear lobes, a high and broad forehead, a short nose, and a thick everted lower lip. Depending on the size and location of the deleted region, patients showed severe intellectual disability (ID) and/or motor impairment. In 2001, Bojinova et al. [8] described an additional 13 patients with cranial anomalies, frontal bossing, broad cheeks, and large ears. Additional case reports of 13q deleted patients involving band 13q14 reported macrocephaly, hypertelorism, proptosis, cleft palate, macroglossia, hypotonia, and mild to severe developmental delay [9,10]. In 2007, Caselli et al. [1], by array-CGH, identified two deleted patients who showed variable clinical features including craniofacial dysmorphism, psychomotor delay, hypotonia, short stature, and anomalies of feet and brain. In 2011, Mitter and colleagues [11] collected and reported clinical, cytogenetic, and molecular data of 63 patients with isolated or familial RB who carried an interstitial 13q deletion involving *RB1*. Patients with a small deletion (within 13q14 and smaller than 6 Mb) displayed macrocephaly, tall stature, obesity, motor, and/or speech delay [11]. Patients with a medium deletion (within 13q12.3q21.2 and sized between 6–20 Mb) showed characteristic facial features, mild to moderate psychomotor delay, short stature, and microcephaly [11]. Patients with a large deletion (within 13q12q31.2 and larger than 20 Mb) had characteristic craniofacial dysmorphisms, mild to severe psychomotor delay, hypotonia, constipation, and feeding problems. Additional features included deafness, seizures, and brain and heart anomalies [11].

13q deletion syndrome involving *RB1* is a rare contiguous gene deletion condition whose genotype-phenotype correlations are still a question of debate. In order to explain ID, Caselli et al. [1] suggested a critical region in 13q14, containing at least 30 genes, among which four genes were considered as good functional candidates for neurodevelopmental delay: *NUFIP1* (nuclear fragile X mental retardation protein 1), *HTR2A* (serotonin receptor 2A), *PCDH8* (protocadherin 8), and *PCDH17* (protocadherin 17). Mitter et al. [11] suggested that heterozygous loss of *NUFIP1* and *PCDH8* may contribute to psychomotor delay, deletion of *MTLR1* to microcephaly, and loss of *EDNRB* to feeding difficulties and deafness [11].

In the present study, we describe seven new patients with 13q deletion involving *RB1* and discuss genotype-phenotype correlation. Array-CGH analysis was performed in all patients, and results showed medium and large deletions of the 13q chromosome, ranging from 13q13.2 to 13q22.3. Overlapping of the deleted regions allows furthering the current knowledge, identifying a new minimal critical region for psychomotor delay, and excluding previously suggested candidate genes.

## 2. Materials and Methods

### 2.1. Human Subjects

Patients came to the Medical Genetics department after been evaluated and hospitalized by the Unit of Ophthalmology and Retinoblastoma Referral Center, and the Unit of Pediatrics, Department of Maternal, Newborn and Child Health (University of Siena, Policlinico “Santa Maria alle Scotte”). For each patient genetic counseling was performed in order to collect family history and better define facial and physical characteristics. All patients’ parents gave their written informed consent to the study that was carried out according to the Declaration of Helsinki.

Genomic DNA was extracted from EDTA peripheral blood samples using MagCore HF16 (Diatech Lab Line), according to the manufacturer’s instructions. The DNA quantity was estimated using the NanoDropTM 2000/2000c Spectrophotometer (ThermoFisher Scientific, Waltham, MA, USA).

### 2.2. Whole-Genome Array-Based Comparative Genomic (a-CGH)

High-resolution whole-genome array-based Comparative Genomic Hybridization (a-CGH) analysis was performed on genomic DNA of the patients and their parents, using the SurePrint G3 Human CGH Microarray 8 × 60k (Agilent Technologies, Santa Clara, CA, USA), a dual-color array containing 60-mer high-quality probes with 41 Kb genome-wide median probe spacing. Copy Number Variants (CNVs) were analyzed and mapped using the Human Genome Assembly GRCh37/hg19.

Slides were scanned using an Agilent G2565CA Microarray Scanner (Agilent Technologies, Santa Clara, CA, USA) and processed using Feature Extraction software (v10.5.1.1). Results were analyzed using Agilent CytoGenomics software (v5.1) with default settings. The results included imbalances with at least three consecutive probes with abnormal log2 ratios. The Database of Genome Variants (DGV- http://dgv.tcag.ca/dgv/app/home; March 2021), DECIPHER (DatabasE of Chromosomal Imbalances and Phenotypes using Ensembl Resources- https://www.deciphergenomics.org/; April 2021), PubMed (https://pubmed.ncbi.nlm.nih.gov; April 2021), UCSC genome browser (https://genome.ucsc.edu; April 2021), Database of Human CNVs (http://gvarianti.homelinux.net/gvariantib37/index.php; March 2021), SFARI (Simon’s Foundation Autism Research Initiative) Gene Database (https://gene.sfari.org; March 2021), and OMIM (Online Mendelian Inheritance in Man- https://www.omim.org/; April 2021) databases were used in the interpretation of the results. Each DNA sample was analyzed twice through a-CGH, in order to confirm the obtained data.

## 3. Results

### 3.1. Clinical Description

Clinical findings of each patient are described in Table 1 and Table 2. Diagnosis of RB was performed in all patients within the first year of life, except in Patient 2 who received an RB diagnosis at 3 years of age. Three patients did not show developmental delay over time (as confirmed by subsequent follow-up). Dysmorphic features were observed except for Patient 1 (Table 1 and Table 2). 

#### 3.1.1. Patient 1 (#349/19)

We first visited patient 1 when she was 4 years old (Table 1). She was the second child of healthy unrelated parents. Family history was silent for RB or other types of tumors. She was born from spontaneous delivery at 30 weeks of gestation, due to premature rupture of membranes. Birth weight was 1.5 kg (50°–75° percentile), length was 46 cm (>90° percentile) (Table 1); for the first month of life, the baby required assistance and remained in the incubator, whereas valid suction was reported (Table 1). At five months, unilateral right RB was diagnosed, firstly treated with chemotherapy and laser therapy with a reduction of the mass. Then, because of the tumor’s relapse, she again performed chemotherapy and laser therapy and finally enucleation (Table 1). The contralateral eye’s controls were always normal. Brain MRI revealed the presence of frontal capillary angioma (Table 2). Normal psychomotor development was reported with first words at 1 year and independent walking between 12 and 18 months (Table 1).

At 4 years old, her weight was 17 kg (50°–75° percentile); height was 101 cm (25°–50° percentile), and Occipital Frontal Circumference (OFC) was 49.5 cm (25°–50° percentile) (Table 1). No peculiar facial features were appreciated (Table 3).

At 14 years old, the girl came back to our attention. Her parameters were: OFC 53.5 cm (36° percentile), height 152.5 cm (12° percentile). Her parents reported good school performance (Table 1).

#### 3.1.2. Patient 2 (#82/2013)

Patient 2 is a Greek female and she was evaluated for the first time at 3 years and 11 months old. (Table 1). The parents’ family history was unremarkable.

In the context of prenatal diagnostic testing, the karyotype identified a pericentric inversion of chromosome 9, inv(9) (p11q13). Prenatal and postnatal periods were normal. Auxological parameters at birth were not available. Normal psychomotor development was reported: babbling at 6–7 months and independent walking at 15 months (Table 1). At the time of the examination, the patient spoke Greek very well and understood enough Dutch. At 3 years and 6 months of age, bilateral RB was diagnosed involving predominantly the right eye, for which enucleation was required, and at 70% the left eye, treated with chemotherapy (Table 1). A physical examination, at 4 years old, showed a length of 103 cm (25°–50° percentile), a weight of 17 kg (50°–75° percentile), and OFC of 51cm (25°–50° percentile) (Table 1). She presented prominent frontal bossing, broad and wide forehead, deeply set eyes, bilateral overfolded helix, and broad nasal bridge with hypoplastic nostrils (Table 2). Clinicians who had taken care of the patient over time reported her last follow-up at 12 years of age without any important problems or learning difficulties, normal growing, and regular social life (Table 1).

#### 3.1.3. Patient 3 (#2148/18)

We first visited patient 3 when she was 5 months old. She was born at term, without any problems during pregnancy. Parents reported previous recurrent miscarriages; however, their karyotype was normal. The growth parameters at birth were in the high normal range (length 52 cm, 85°–97° percentile; weight 4.0 kg, 85°–97° percentile; and OFC 38 cm, 95°percentile) (Table 1). At 4 months, unilateral left RB was diagnosed, and enucleation was needed (Table 1). A physical examination, at 1 year and 5 months, showed a weight of 11.5 kg (25° percentile), and OFC of 51cm (>99° percentile) (Table 1). She presented a wide and broad forehead, prominent frontal bossing, bushy eyebrows in the third medium and sparse in the lateral third, hypertelorism, deeply set eyes, depressed nasal root, broad nasal tip, everted nostrils, prominent filter, Cupid’s bow, and everted lower lip (Table 2). The patient walked independently, acquired fine motor skills, and pronounced a few words (Table 1). Clinicians reported that at the age of three the patient used good language, while sphincter control was not yet acquired (Table 1).

#### 3.1.4. Patient 4 (#2794/20)

Patient 4 was a 9-month-old female, the second of healthy and unrelated parents (Table 1). The family history was negative for other cases of RB and other ocular problems. On the paternal side, there were many relatives with different types of tumors. She was born at 39 weeks of gestation by cesarean section, because of maternal scoliosis. Pregnancy was complicated by hypertension and intrauterine growth retardation, in absence of flowmetry alterations. Growth parameters at birth were: length 47 cm (15°percentile); weight 2.25 kg (<5° percentile) (Table 1). Hypovalid suction and reflux were reported during the perinatal period. At 9 months, unilateral right RB was diagnosed and chemotherapy was required (Table 1).

A physical examination at nine months showed a length of 63 cm (<5° percentile), a weight of 6.63 kg (75°–90° percentile), and OFC of 38.8 cm (<5° percentile). No sitting position was acquired; the patient presented bushy eyebrows, wide forehead, small eyes with apparent hypertelorism, broad nasal bridge, long and prominent filter, low-set posteriorly rotated ears (Table 2).

At 1 year and 3 months, the patient showed important neuromotor delay (Table 1). She acquired sitting position and fine motor skills; babbling was absent, no crawling or walking movements were observed. Highlighted only supine to prone rolling (Table 1).

#### 3.1.5. Patients 5–6 (#387–#404)

At first evaluation, patients 5 and 6 were 6 months old female monozygotic twins; they were born at 37 weeks of gestation by cesarean section. The parents’ family history was unremarkable. Patient 5 growth parameters at birth were: length 42 cm (<1° percentile); weight 2.04 kg (<1° percentile); OFC 31.5 cm (10–25°percentile) (Table 1). At the time of the evaluation, she had not totally acquired head control, and she was hospitalized for a seizure episode. At five months, unilateral bifocal left RB was diagnosed, treated with systemic and intra-arterial chemotherapy (Table 1). The MRI showed “slight dilatation of the supratentorial ventricular system in relation to partial agenesis of the corpus callosum”; echocardiography showed: “3 mm interatrial defect, with left > right shunt and slight dilation of right chambers’’ (Table 2). A physical examination, at 6 months, showed a length of 62 cm (25° percentile), a weight of 5.16 kg (<5° percentile), and OFC of 39.8 cm (3–10° percentile) (Table 1). She presented asymmetrical brachycephaly, wide forehead, well-defined philtrum, thin upper lip, clinodactyly of 3rd–5th toes, and cutis marmorata (Table 2).

At birth, patient 6 parameters were: 42 cm for length (<1° percentile) and 1.95 kg for weight (<1° percentile). At six months, physical examination highlighted brachycephaly, OFC 40 cm (3°–10° percentile), wide forehead, well-defined designed philtrum, thin upper lip, clinodactyly of toes, and cutis marmorata. At 10 months, right RB was diagnosed, then treated with brachytherapy (Table 1).

At 7 years old, both showed an important psychomotor development delay: language was not still acquired, nor independent walking or sphincter control (Table 1). They both showed important stereotyped hand movements along the midline and movements of the trunk (Table 1).

At 13 years old, both had not yet acquired any language skills, nor independent walking. Hands stereotypes were no longer present; their sexual development was normal (Table 1).

#### 3.1.6. Patient 7 (#3311/17)

Patient 7 was an 8-month-old male, the second child of healthy and unrelated parents. The family history was silent for ocular problems and tumors. He was born at 36 weeks of gestation; his pregnancy was complicated by oligohydramnios during the last trimester. Growth parameters at birth were: length 45 cm (>25° percentile), weight 2.095 kg (10° percentile), and OFC 32.9 cm (50° percentile) (Table 1). Absent gaze and strabismus were noticed since the first days of life. A neurological examination reported: reduced motor findings, axial hypotonus, poor reactivity to auditory stimuli. The V.E.P (Visual Evoked Potential) found possible bilateral sensorineural hearing loss.

At 7 months, bilateral RB was diagnosed, then treated with chemotherapy (Table 1). Brain-MRI showed “Plagiocephaly and hypoplastic brain stem”. Growth parameters at 8 months were in the normal range (length 60 cm, 50° percentile; weight 5.8 kg, 25° percentile (Table 1). He presented global developmental delay and hypotonia, poor head control, absent babbling (Table 1). Facial features included frontal bossing, wide and broad forehead, hypertrichosis, bushy eyebrows in the lateral third, low-set ears, depressed nasal root, prominent filter, slight micrognathia, and prominent lower lip (Table 2).

At 2 years old, clinicians reported axial hypotonia, poor head control, and motor skills. Sitting position was not acquired, and language was still absent; it was reported only liquid feeding because of chewing difficulties (Table 1).

### 3.2. Molecular Characterization

**Figure 1:**
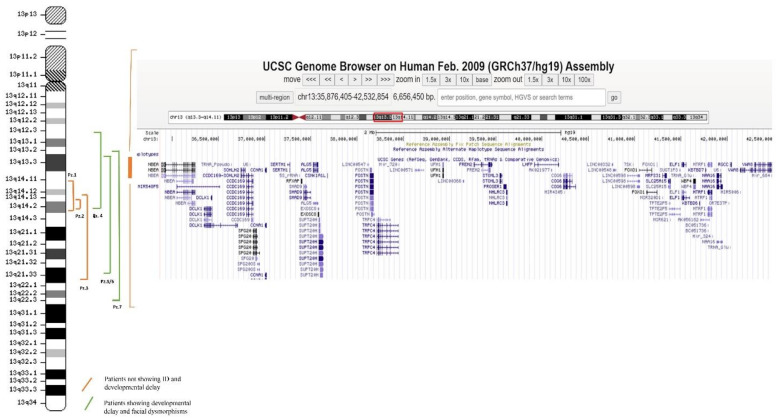
Minimal critical region for ID and developmental delay. The figure shows the deletions reported in all the patients involved in the study. Orange lines refer to patients not showing ID and developmental delay; green lines refer to patients showing developmental delay and facial dysmorphisms. Overlapping the CNVs found in these last patients allowed defining a new minimal critical region for ID which contains the 36 genes mentioned in the text.

Results obtained by Array-CGH are summarized in Table 3; Copy Number Variants (CNVs) breakpoints all refer to the Human Genome Assembly GRCh37/hg19. The analysis revealed medium and large deletions of the 13q chromosome, spanning from 13q12.3 to 13q22.3. Segregation analysis in parents of all patients except Patient 2 showed the de novo origin of each deletion. The analysis did not identify any additional CNV in the whole cohort. In order to identify a minimal critical region for developmental delay, we excluded from our analysis the regions found in Patients 1,2,3, who did not show global developmental delay or ID, and we overlapped the rearrangements of the remaining patients. The study allowed us to focus on cytobands 13q13.3q14.11 (Figure 1). The new minimal critical region contains 36 key genes: *ALG5*, *CCDC169*, *CCDC169-SOHLH2*, *CCNA1*, *COG6*, *CSNK1A1L*, *DCLK1*, *ELF1*, *EXOSC8*, *FOXO1*, *FREM2*, *KBTBD6*, *KBTBD7*, *LHFPL6*, *MAB21L1*, *MRPS31*, *MTRF1*, *NAA16*, *NBEA*, *NHLRC3*, *POSTN*, *PROSER1*, *RFXAP*, *RGCC*, *SERTM1*, *SLC25A15*, *SMAD9*, *SOHLH2*, *SPART*, *SPART-AS1*, *STOML3*, *SUPT20H*, *TRPC4*, *UFM1*, *VWA8*, and *WBP4* (Figure 1). Among these, five are genes already reported to play a role in ID (Table 4).

## 4. Discussion

Patients with a 13q deletion containing *RB1* show a variable phenotype that, depending on the size and location of the deleted region, includes ID, growth retardation, craniofacial dysmorphisms, congenital malformations, and increased risk of retinoblastoma. In the present study, we characterized by array-CGH seven new cases with 13q deletion involving *RB1* and discussed genotype-phenotype correlations.

The overlap of the seven deleted regions allows us to go further the current knowledge, identifying a new minimal critical region for psychomotor delay containing 36 genes: *ALG5*, *CCDC169*, *CCDC169-SOHLH2*, *CCNA1*, *COG6*, *CSNK1A1L*, *DCLK1*, *ELF1*, *EXOSC8*, *FOXO1*, *FREM2*, *KBTBD6*, *KBTBD7*, *LHFPL6*, *MAB21L1*, *MRPS31*, *MTRF1*, *NAA16*, *NBEA*, *NHLRC3*, *POSTN*, *PROSER1*, *RFXAP*, *RGCC*, *SERTM1*, *SLC25A15*, *SMAD9*, *SOHLH2*, *SPART*, *SPART-AS1*, *STOML3*, *SUPT20H*, *TRPC4*, *UFM1*, *VWA8*, and *WBP4.* Among the deleted genes, *NBEA* was selected as the candidate for the impaired neurodevelopmental phenotype.

Among the deleted genes, five of them were selected because of their involvement in impaired neurogenesis, when mutated (Table 4). In particular, *NBEA* encodes for Neurobeachin, a brain-specific kinase-anchoring protein implicated in vesicle trafficking and synaptic structure and function [12,13,14,15]. The gene is reported mutated in neurodevelopmental disorder with or without epilepsy [16] and it was cited with a score of 1 in the SFARI Gene database as a candidate gene for autism spectrum disorder (data referred to the latest SFARI Gene database update, June 20th, 2019) [17]. Studies on the murin Nbea gene, which shares high homology to human *NBEA* [18], established the involvement of Nbea in synaptic transmission and exocytosis [19], whereas other animal studies suggested a role in neuronal excitability [20] and brain development [21].

Concerning the remaining genes, *MAB21L1*, *DCLK1*, *EXOSC8*, and *SPART* haploinsufficiency might contribute to the observed psychomotor delay. *MAB21L1* is contained within the neurobeachin gene [22] and belongs to the conserved male abnormal gene family 21 (Mab21) [23]. Mab21 was first described in the nematode *Caenorhabditis elegans* as a transcription factor in cell fate determination [24]. Mab21 genes play a major role in embryonic development, but gene expression extends beyond the developmental period well into adulthood [25]. Biallelic *MAB21L1* loss-of-function mutations cause an extremely rare autosomal recessive recognizable condition, the Cerebellar, Ocular, cranioFacial, and Genital syndrome (COFG syndrome), which could be reported in association with moderate-to-severe developmental delay/ID and behavioral abnormalities [23]. *DCLK1* was originally identified as a causative gene of cerebral cortical malformation such as lissencephaly [26,27] and was later found to regulate neuronal migration and axon outgrowth [26,27,28,29,30,31]. *DCLK1* exerts multiple roles in neurogenesis, neuronal migration, axon/dendrite growth, and spine formation [32,33,34,35]. Recent genome-wide association studies (GWAS) and transcriptome analysis indicate that the *DCLK1* gene might be a candidate gene for neurodevelopmental and neuropsychiatric disorders [36,37]. *EXOSC8* is a member of the RNA exosome, a conserved multi-protein complex that is essential for correct RNA processing and degradation [38,39]. Recessive variants in exosome components *EXOSC3*, *EXOSC8*, and *RBM7* cause various constellations of pontocerebellar hypoplasia (PCH), spinal muscular atrophy (SMA), and central nervous system hypomyelination [40,41]. Exosomal proteins are important not only for neuronal development but also for the survival of spinal motor and cerebellar neurons [42]. *SPART* is the pathogenic gene causing Troyer Syndrome, an extremely rare autosomal recessive disease that affects the nervous system, characterized by spastic paraplegia and distal amyotrophy [43,44]. Spartin has different expression levels in various tissues and organs, and it is highly expressed in the temporal lobe, anterior central gyrus, corpus callosum, adipose tissue, and the placenta [45,46].

Concerning craniofacial dysmorphisms, in our group of patients, a broad and wide forehead was reported in 85.7% (6/7); a prominent filter in 71.4% (5/7); ear abnormalities (everted or folded helix, low-set/everted earlobes), frontal bossing, bushy eyebrows, deep-set or small eyes, thick everted lower lip, brain anomalies, and altered head morphology in 43% (3/7); depressed nasal root, everted or hypoplastic nostrils, broad nasal bridge, clinodactyly, and cutis marmorata in 28.5% (2/7). Among other less common features, hypertrichosis, micrognathia, cardiac defects, and hearing loss were observed in 14.2% of the cases (1/7). With the exception of Patient 1, who did not show any particular trait, our data overall confirm a previous correlation between facial dysmorphisms/congenital anomalies and the 13q12.3q31 region, identifying it as a critical region related to these broad phenotypic spectra [11].

In conclusion, this study adds novelties regarding the genotype-phenotype correlation in 13q deletion syndrome including *RB1*, defining a new key region for developmental delay. The focus on the 13q13.3q14.11 region allowed us to exclude the possible involvement of the previously reported candidate genes *HTR2A*, *NUFIP1*, *PCDH8*, and *PCDH17* in ID and global psychomotor delay development [1]. Further studies are however needed to characterize the contribution of different genes involved and to understand the impact of the haploinsufficiency of this region on ID. Moreover, as more patients with overlapping deletion will be identified, more precise conclusions on genotype/phenotype correlation can be drawn.

It is well ascertained that neurodevelopmental disorders can arise from many different genetic abnormalities ranging from chromosomal and genomic defects to single nucleotide variations in single genes. The array-CGH performed in our case series did not identify any additional CNV possibly accounting for the neurodevelopmental phenotype of our patients. However, it cannot be ruled out that smaller CNVs or single-nucleotide variations undetectable by array-CGH may act as cofactors for ID.

## 5. Conclusions

The study provides new clinical and molecular information useful for the management of cases with a 13q deletion, especially in the prenatal and neonatal settings. A better understanding of the genotype/phenotype correlation can enable clinical geneticists to provide more definite prognostic information to couples who receive a prenatal diagnosis of 13q deletion. Furthermore, our data reinforce the idea that once 13q deletion syndrome diagnosis is established, a multidisciplinary approach is needed by a team of specialists, including, besides the ophthalmologist, pediatric oncologist, pathologist, and radiation oncologist, also the neuropsychiatrist, the speech therapist, and the psychomotor therapist, in order to fully manage all the different aspects of the syndrome and their progression over time

## Figures and Tables

**Table 1:** Clinical features of patients 1–7.

Features	Patient 1	Patient 2	Patient 3	Patient 4	Patient 5 (Twin of Patient 6)	Patient 6 (Twin of Patient 5)	Patient 7
**Sex**	F	F	F	F	F	F	M
**Age (years/months) at first examination**	4 years	3 years and 11 months	5 months	9 months	6 months	6 months	8 months
**Auxological parameters at birth (OFC, weight, length)**	Weight 1.50 kg (50°–75° percentile); length 46 cm (>90° percentile)	n.a.	OFC 38 cm (95° percentile); weight 4.0 kg (85°–97° percentile); length 52 cm (85°–97° percentile)	Weight 2.25 kg(<5° percentile); length 47 cm (15° percentile)	OFC 31.5 cm (10°–25° percentile); weight 2.04 kg (<1° percentile); length 42 cm (<1° percentile)	Weight 1.95 kg (<1° percentile); length 42 cm (<1° percentile)	OFC 32.9 cm (50° percentile); weight 2.075 kg (10° percentile); length 45 cm (>25° percentile)
**Auxological parameters at evaluation (OFC, weight, length)**	OFC 49.5 cm (25°–50° percentile); weight 17 kg (50°–75° percentile); height 101 cm (25–50° percentile)	OFC 51 cm (25°–50° percentile); weight 17 kg (50°–75°percentile); height 103 cm (25°–50° percentile)	OFC 51 cm (>99° percentile); weight 11.5 kg (25° percentile)	OFC 38.8 cm (<5° percentile); weight 6.63 kg (75°–90° percentile); height 63 cm (<5° percentile)	OFC 39.8 cm (3°–10° percentile); weight 5. 16 kg (<5° percentile); height 62 cm (25° percentile)	OFC 40 cm (3–10° percentile)	Weight 5.8 kg (25° percentile); height 60 cm (50° percentile)
**RB characteristics (age at diagnosis, tumor)**	5 months; unilateral, left eye	3 years old; bilateral	4 months; unilateral, left eye	9 months; unilateral, right eye	6 months; unilateral, left eye	6 months; unilateral, left eye	7 months; bilateral
**RB treatment**	Chemotherapy and laser therapy; enucleation	Enucleation of the right eye; chemotherapy of the left eye	Enucleation	Chemotherapy	Systemic and intra- arterial chemotherapy	Brachytherapy	Chemotherapy
**Facial dysmorphisms (yes/no)**	No	Yes	Yes	Yes	Yes	Yes	Yes
**Developmental psychomotor delay (yes/no)**	No	No	No	Yes	Yes	Yes	Yes
**Follow up (age in years; considerations)**	14 years and 9 months; good school performance, no specific facial features, denied further major diseases. No ID or developmental delay.	12 years; no developmental delay, very good school performance, good social integration, normal performance in sports.	3 years; independent walking, acquired fine motor skills. Good language skills.	1 year and 3 months: neuromotor delay. Acquired sitting position, hands folded but fine motor skills. Absent babble speech, no crawling or walking, but supine to prone rolling.	7 years; important psychomotor delay. No language skills, not acquired sphincter control. Walking only with support. Important hand stereotypies on the midline, trunk movements. 13 years: no language skills, nor independent walking. No hands stereotypes anymore; normal sexual development.	7 years; important psychomotor delay. No language skills, not acquired sphincter control, walking only with support. Important hand stereotypies on the midline, trunk movements. Important difficulties in walking. 13 years: no language skills, nor independent walking. No hands stereotypes anymore; normal sexual development.	2 years; axial hypotonia, poor head control. No sitting position, absent language. Exclusively liquid feeding.

M = Male; F = Female; OFC = Occipital Frontal Circumference; RB= Retinoblastoma; n.a.= not available.

**Table 2:** Dysmorphic features and clinical findings in patients 1–7. “+”: present; “−“: absent.

Dysmorphic Features	Patient 1	Patient 2	Patient 3	Patient 4	Patient 5 (Twin of Patient 6)	Patient 6 (Twin of Patient 5)	Patient 7
**Broad and wide forehead**	−	+	+	+	+	+	+
**Frontal bossing**	−	+	+	−	−	−	+
**Bushy eyebrows**	−	−	+	+	−	−	+
**Deeply-set or small eyes**	−	+	+	+	−	−	−
**Nostrils anomalies**	−	+; hypoplastic	+; everted	−	−	−	−
**Prominent philtrum**	−	−	+	+	+	+	+
**Depressed nasal root**	−	−	+	−	−	−	+
**Broad nasal bridge**	−	+	−	+	−	−	−
**Thick everted lower lip**	−	−	+	−	+	+	−
**Ear abnormalities**	−	+; overfolded helix	−	+; low-set posteriorly rotated ears	−	−	+; low-set
**Others**							
**Hypertrichosis**	−	−	−	−	−	−	+
**Micrognathia**	−	−	−	−	−	−	+
**Altered head morphology**	−	−	−	−	Brachycephaly	Brachycephaly	Plagiocephaly
**Clinodactyly**	−	−	−	−	+	+	−
**Cutis Marmorata**	−	−	−	−	+	+	−
**Brain anomalies**	Frontal capillary angioma	−	−	−	Agenesis of the corpus callosum	−	Brain stem hypoplasia
**Cardiac anomalies**	−	−	−	−	3 mm interatrial defect, with left > right shunt and slight dilation of right chambers	−	−
**Hearing loss**	−	−	−	−	−	−	+; sensorineural, bilateral

**Table 3:** Molecular results obtained by Array- CGH in chromosome 13. Copy Number Variants (CNVs) breakpoints all refer to the Human Genome Assembly GRCh37/hg19. De novo: present only in the proband.

Features	Patient 1	Patient 2	Patient 3	Patient 4	Patient 5 (Twin of Patient 6)	Patient 6 (Twin of Patient 5)	Patient 7
**Origin of deletion/heredity**	de novo	n.a.	de novo	de novo	de novo	de novo	de novo
**Array CGH analysis (proximal-distal breakpoints of the deletion)**	42532854_50275341	46851293_49614283	45712553_71933242	30898736_49309890	35876405_69983996	35876405_69983996	35398085_75462802
**Location and size of deletion**	13q14.11q14.2; 7.8 Mb	13q14.14q14.2; 2.76 Mb	13q14.12q21.33; 26.24 Mb	13q12.3q14.2; 18.4 Mb	13q13.2q21.33; 34.1 Mb	13q13.2q21.33; 34.1 Mb	13q13.2q22.3; 40 Mb

n.a. = not available. Segregation analysis not performed.

**Table 4:** Genes reported to play a role in ID.

Gene Symbol (OMIM#).	Phenotype (OMIM#)	Inheritance	Association with Neurodevelopmental Disorders
*NBEA* (OMIM#604889)	Neurodevelopmental disorder with or without early-onset generalized epilepsy (OMIM#619157)	AD	Reported mutated in autosomal dominant neurodevelopmental disorder with or without epilepsy [12]. It was cited with a score of 1 in the SFARI Gene database as a candidate gene for autism spectrum disorder [13]. It is involved in synaptic transmission and exocytosis [14], neuron excitability, and brain development [15].
*MAB21L1* (OMIM#601280)	Cerebellar, ocular, craniofacial, and genital syndrome (OMIM#618479)	AR	Loss-of-function mutations cause an extremely rare autosomal recessive condition, the Cerebellar, ocular, craniofacial, and genital (COFG) syndrome, characterized by moderate to severe developmental delay and impaired intellectual development [16].
*DCLK1* (OMIM#604742)	Association with neurodevelopmental and neuropsychiatric disorders	N/A	The gene exerts multiple roles in neurogenesis, neuronal migration, axon/dendrite growth, and spine formation [17,18,19,20]. GWAS indicated an association between *DCLK1* and neurodevelopmental and neuropsychiatric disorders [21].
*EXOSC8* (OMIM#606019)	Pontocerebellar hypoplasia, type 1C (OMIM#616081)	AR	Patients with recessive *EXOSC8* mutations present with a characteristic spectrum of overlapping phenotypes of infantile onset hypomyelination, cerebellar and corpus callosum hypoplasia and spinal muscular atrophy [22,23].
*SPART* (OMIM#607111)	Troyer syndrome (OMIM#275900)	AR	Causative gene of the Troyer Syndrome, a rare autosomal recessive disease characterized by spastic paraparesis, developmental delay, dysarthria, distal amyotrophy, skeletal defects, and short stature [24,25].

AD: Autosomal Dominant; AR: Autosomal Recessive; N/A: not applicable.

## Data Availability

Data sharing is not applicable to this article as no datasets were generated or analysed during the current study.
